# Neurocognitive effects of interest on reward valuation and effort investment in boring contexts

**DOI:** 10.3758/s13415-026-01403-7

**Published:** 2026-02-14

**Authors:** Dajung Diane Shin, Sun Kyung Lee, Yi Jiang, Jeesoo Lee, Jae Hyung Ahn, Sung-il Kim

**Affiliations:** 1 Department of Education, Kyungnam University, Changwon, Korea; 2 People Analytics Lab, Samsung Electronics (DS), Seoul, Korea; 3 Department of Educational Psychology, East China Normal University, Shanghai, China; 4 Open Major Division (Division of Interdisciplinary Studies), Hankuk University of Foreign Studies, Seoul, Korea; 5 Department of Behavioral Psychology, Kennedy Krieger Institute, Johns Hopkins School of Medicine, Baltimore, MD USA; 6 Department of Education and the Brain & Motivation Research Institute (bMRI), Korea University, Seoul, Korea

**Keywords:** Effort, Interest, Reward, Incentive, Replenishment, fMRI

## Abstract

Monotonous tasks are common in academic and professional settings, yet sustaining motivation and effort in such contexts remains a persistent challenge. This research examined whether inserting a brief episode of interest into an otherwise boring, incentivized task could replenish cognitive and motivational resources and sustain effort. Two competing accounts were tested. The contrast-effect hypothesis predicted that once interest was withdrawn, the remaining rewards would lose their impact, and effort would diminish. In contrast, the transfer-effect hypothesis proposed that prior interest would leave a motivational trace that preserves the effectiveness of the rewards. Study 1 (behavioral study) employed a typing task, while Study 2 (fMRI study) used a social preference guessing task, both with performance-contingent monetary incentives. In both studies, participants in the experimental group completed a boring–interesting–boring sequence, whereas those in the control group experienced a boring–neutral–boring sequence. Across two studies, results consistently supported the transfer-effect hypothesis, demonstrating that prior interest enhanced subsequent engagement, effort, and reward sensitivity, even when monetary incentives were reduced. Neuroimaging results further revealed activity in regions involved in reward processing (e.g., anterior cingulate cortex, anterior insula) and attentional control (e.g., lateral occipital cortex, inferior parietal lobule) even after interest was removed, suggesting a lingering motivational effect of interest. These findings integrate reinforcement learning and interest theories and suggest a practical strategy: sandwiching brief, interest-enhancing episodes between boring rewarded tasks can serve as a motivational reset, enabling effort and persistence in low-stimulation environments.

**Supplementary Information:**

The online version contains supplementary material available at 10.3758/s13415-026-01403-7.

## Introduction

Imagine working late one night, tasked with manually transcribing printed pages, word by word, line by line. At first, the promise of a paycheck keeps you going. But soon, the monotony sets in, making it difficult to sustain focus and effort. Your mind drifts, your fingers slow, and each sentence feels heavier than the last. Then, unexpectedly, your eyes land on a paragraph that captivates you—a story, an idea, a phrase that resonates with your personal interests. In that moment, the task feels lighter, almost effortless, and your pace quickens. This fleeting spark of interest not only restores your energy but also rekindles your willingness to persist. Such moments illustrate a broader psychological phenomenon: Even a brief moment of interesting engagement can temporarily shift attention away from incentives, replenish self-regulatory resources, and sustain effort.

Boring tasks present a persistent challenge across domains such as education, work, sports training, and rehabilitation. Because humans are naturally inclined to seek pleasure and avoid discomfort, boredom—characterized by low arousal and disengagement—can markedly undermine persistence and performance (Eastwood et al., 2012; Fisher, 1993). Two widely used countermeasures are the provision of rewards and induction of interest in the task (e.g., Hamner & Foster, 1975; Sansone et al., 1992; Weibel et al., 2010). The most favorable motivational state likely arises when both operate within the same task. Rewards (e.g., pay, points) offer immediate, tangible returns that jump-start engagement and boost short-term performance, partly by recruiting dopaminergic circuitry central to reward prediction and expectation (Bardach & Murayama, 2025; Cameron & Pierce, 1994; Knutson & Cooper, 2005; Liu et al., 2011; Schultz, 2016). Interest, by contrast, is enjoyment inherent to the activity. It supports attention and cognitive efficiency, offsets fatigue with positive affect, and scaffolds self-regulation over time (Hidi & Renninger, 2006; O’Keefe & Linnenbrink-Garcia, 2014; Silvia, 2008; Song et al., 2019). Whereas rewards rely on anticipated outcomes, interest is rooted in the activity itself, making it a powerful driver of long-term participation and deep learning.

But what happens when only one, either reward or interest, is available in a task, or when one is transient? Prior work has largely focused on the effects of introducing and subsequently withdrawing rewards from an interesting task. These studies have consistently found that rewards can depress later motivation and effort relative to never rewarded baselines, a phenomenon known as the undermining effect (Deci, 1971; Lepper et al., 1973; Weibel et al., 2010). The reverse sequence—briefly adding interest to an otherwise uninteresting yet rewarded task—has received far less attention, particularly under genuinely boring conditions. The present study addressed this gap. We examined whether *sandwiching* a short, interest-eliciting episode between two blocks of an otherwise monotonous, rewarded task confers a lasting motivational benefit or, upon its removal, precipitates a comparable decline. We tested this in a two-part investigation: a behavioral experiment (Study 1) and an fMRI investigation (Study 2). By integrating motivational theory with cognitive neuroscience, we sought to clarify how reward and interest interact over time to sustain effort in low-stimulation contexts.

### When an interesting task meets incentives: Explanations for motivational trade-offs

Traditionally, the rewards–interest relation has been framed within an extrinsic–intrinsic motivation dichotomy. However, Kim (2025) challenged this binary view, arguing that both rewards and interest engage the same dopaminergic circuitry in the brain (Adcock et al., 2006; Di Domenico & Ryan, 2017; Kang et al., 2009; see also Bardach & Murayama, 2025). This system encodes reward value by integrating features such as reward type, salience, magnitude, contingency, and required effort (Hare et al., 2008; Knutson et al., 2005; Salamone & Correa, 2024; Schultz, 2006). In practice, motivation is rarely purely extrinsic or purely intrinsic; the relative contributions of rewards and interest can coexist, shift over time, and depend on how rewards are construed (e.g., controlling vs. self-endorsed). Consequently, distinguishing between incentives (valued as a means to an end) and interest (valued for the activity itself) may offer a more functionally meaningful framework for understanding motivation than the traditional extrinsic–intrinsic divide (Kim, 2025).

In real-world learning and work contexts, incentives and interest often coexist but frequently decouple: One endures while the other gradually diminishes or disappears (e.g., no longer earning bonus points or losing interest in the activity). Much of the existing research has examined the undermining effect, focusing on how removing incentives from initially interesting tasks diminishes subsequent engagement and effort (Deci, 1971; Lepper et al., 1973). Using fMRI, Murayama et al. (2010) showed that performance-contingent monetary rewards for interesting tasks transiently elevated activation in dopaminergic regions, including the bilateral striatum and midbrain. Once the rewards were withdrawn, this activation dropped sharply, and the decline was significantly associated with reduced voluntary effort. Complementing this with EEG, Ma et al. (2014) reported that rewards diminished individuals’ sensitivity to performance outcomes; after financial incentives were removed, the feedback-related negativity (FRN)—a marker of motivational salience—showed a reduced success–failure differentiation. Collectively, these findings indicate that while monetary rewards can transiently upregulate motivational systems, their withdrawal can attenuate reward coding and erode sustained engagement.

The undermining effect has been typically explained by overjustification. In this view, when a strong and salient incentive is introduced, behaviors initially performed for the inherent enjoyment of the task become reattributed to the pursuit of that incentive. The individuals thus come to view the activity not as enjoyable but as a means to obtain a reward (Deci, 1971; Lepper et al., 1973). This shift in attribution or perceived locus of causality weakens intrinsic motivation. Once the reward is withdrawn, the internal justification for engaging in the task diminishes, leading to lower persistence, diminished performance, and decreased willingness to reengage. However, this explanation has been challenged as undermining-like decreases also appear under conditions that preclude explicit self-attribution (e.g., reward downshifts in nonverbal animals), suggesting that attributional processes may not be necessary condition for the effect (Zentall, 2013; see also Hidi, 2016; Murayama, 2022). Complementing this point, neuroimaging evidence suggests that undermining may involve changes in valuation and salience systems that are not readily captured by an overjustification account.

An alternative account for the undermining effect is the contrast effect (Hidi, 2016; Zentall, 2005), which holds that the subjective value of a task is judged relative to recent reward history. Building on Flaherty’s research on incentive relativity in animal behavior (Flaherty, 1999), this account posits that a downshift in expected incentives produce a disproportionately large disruption in responding. Thus, when a task shifts from a high-reward condition (interest + incentive) to a lower-reward condition (interest alone), its perceived value declines more sharply than in unshifted controls (Papini, 2006). From a neurobiological perspective, this relative drop in value can be understood through the reward prediction error (RPE; Glimcher, 2011; Schultz, 1998). Pairing an incentive with an interesting task elevates the brain’s expected value of performing that task. When the incentive is removed, the discrepancy between this heightened expectation and the actual outcome generates a negative RPE (worse than expected), leading to a sharp reduction in dopaminergic activity. This reduction appears to affect not only core reward circuitry (ventral striatum, ventral tegmental area [VTA]) but also broader motivational networks that sustain persistence (Ma et al., 2014; Murayama et al., 2010). Even if interest remains, the relative loss in the magnitude of reinforcement and resulting negative RPE can amplify psychological disappointment, further undermining motivation (Zentall, 2005).

### When a boring incentivized task meets interest: Two opposing possibilities

While much of the literature has examined how removing incentives affects tasks that remain interesting, far less is known about the reverse scenario. A key open question is whether a comparable process occurs when interest is introduced into an incentivized task that is initially uninteresting or boring, but then fades before the incentive is withdrawn. Understanding how incentives operate after the loss of interest is essential not only for clarifying time-lagged shifts in motivation but also for determining how sustained effort is allocated and maintained under changing motivational conditions. This issue is particularly important in monotonous contexts, where effort regulation often determines both performance quality and persistence. Addressing this gap would refine theoretical models of incentive–interest interaction and inform the design of learning, work, and training environments in which motivational sources and the effort they elicit are dynamic and subject to change.

Two opposing mechanisms may account for motivational changes when interest and incentives shift asynchronously; that is, when interest is temporarily introduced into a boring, incentivized task but disappears before the incentive is removed. The first is the contrast effect. Similar to the mechanism underlying the undermining effect, the contrast between a prior high-reward state (interest + incentive) and a current reduced-reward state (incentive alone) may lead to devaluation of the remaining incentive, reducing both cognitive and behavioral investment. Neurobiologically, the affective reward value associated with the earlier interest + incentive state may persist as an expectation even after interest is gone. When the experienced enjoyment falls short of this expectation, a negative RPE is likely to occur. Although the remaining monetary incentives may still activate dopaminergic pathways, their subjective utility may be diminished relative to the previous high-reward state. As a result, the residual incentive may yield less satisfaction and, in some cases, foster psychological deprivation and reduce willingness to persist.

The second possibility is the transfer effect. This account posits that motivational momentum from the preceding interesting element would reduce the aversiveness of the boring task that follows and replenish mental resources for investing effort and detecting the salience of reward. Interest enhances learning by boosting positive affect and attention, deepening cognitive processing, and improving memory, while also lowering the perceived cost of effort (Ainley et al., 2002; Renninger & Hidi, 2016; Schiefele, 1991; Song et al., 2019). Thus, motivation driven by interest tends to be enduring and self-sustaining (Hidi & Ainley, 2008; Sansone & Thoman, 2005). One of the most compelling functions of interest is its capacity to replenish mental resources, rendering even effortful or monotonous tasks less cognitively taxing. This restorative function is especially critical in contexts characterized by mental fatigue, cognitive overload, or prolonged monotony.

Several lines of evidence support the transfer effect. First, the broaden-and-build theory (Fredrickson, 2001) proposes that positive emotions, such as interest and enjoyment, expand an individual’s cognitive repertoire and behavioral flexibility. By widening the scope of attention and thought–action tendencies, these emotions could build enduring psychological and social resources that outlast the initial emotional state. In the context of a monotonous but incentivized task, an early episode of interest may enhance attentional control, working memory, and cognitive flexibility. This temporary “broadening” effect could persist into subsequent phases of the task, even after the initial source of interest is removed.

Second, empirical findings consistently showed that even brief experiences of interest can restore the mental energy required for self-regulation, enabling individuals to sustain effort on demanding tasks while experiencing less fatigue (Endres et al., 2025; Milyavskaya et al., 2021; O’Keefe & Linnenbrink-Garcia, 2014; Thoman et al., 2011). For example, Thoman et al. (2011) demonstrated through a series of experiments that interest enhanced effort and persistence in subsequent unrelated tasks, even after participants had expended substantial psychological resources on boring and onerous activities. Notably, participants who engaged in an interesting task (e.g., solving mystery passages) showed greater restoration of psychological resources than those in positive (e.g., searching for positive words) or neutral (e.g., searching for neutral words) task conditions, despite the former being more cognitively demanding. The restorative effects of interest appeared to operate independently of positive affect, self-efficacy, and general achievement motivation (Milyavskaya et al., 2021; O’Keefe & Linnenbrink-Garcia, 2014; Thoman et al., 2011).

Although the precise mechanism of this restorative effect remains unclear, it may be partly explained by the neural activation associated with interest. Interest engages regions involved in motivational valuation, cognitive control, and salience detection (Di Domenico & Ryan, 2017; Shin et al., 2022). These included dopaminergic reward-related regions such as the striatum and ventromedial prefrontal cortex (vmPFC), as well as regions implicated in cognitive control (e.g., dorsolateral prefrontal cortex [dlPFC], anterior cingulate cortex [ACC]) and the salience network (e.g., anterior insula [AI], dorsal ACC). Among these regions, the ACC plays a central role in anticipating outcomes and detecting conflicts between expectations and actual outcomes, thereby engaging cognitive control, effort regulation, and value-updating processes to integrate effort and reward information (Matsumoto & Tanaka, 2004; Onoda et al., 2008). The AI, meanwhile, has been identified as a key neural substrate of intrinsic motivation (Di Domenico & Ryan, 2017; Lee & Reeve, 2013; Murayama et al., 2010, 2015) and serves as a core hub of the salience network (Wiech et al., 2010)—an adaptive system that detects motivationally significant signals and allocates attentional and cognitive resources accordingly (Menon & Uddin, 2010). Thus, experiencing interest could attach greater value, control, and salience to the task itself, with these effects possibly carrying over into later task phases and helping to maintain cognitive control and valuation of the task even after interesting engagement has subsided.

Such carryover effects may occur because interest temporarily shifts motivational focus from external factors to the task itself. During interesting engagement, attentional and control systems may be oriented toward the activity rather than the incentives, allowing self-regulatory resources to be replenished and reducing the depletion that typically accompanies prolonged, effortful engagement. When the task reverts to a purely incentivized form, these replenished resources could delay the onset of motivational decline. Moreover, because individuals attend less to incentives while engaged with interesting elements, the perceived value of subsequent rewards may be refreshed, enhancing their salience and preserving positive RPE signals (better than expected) over time. In this way, the transfer effect underscores the complementary interplay, rather than a trade-off, between incentives and interest.

## Current study

The present study examined whether briefly inserting an interesting element into a monotonous, incentivized task would alter subsequent motivation and effort once that element was removed and, if so, through which mechanisms interest exerts its effects during this transition. We considered two competing explanations. The contrast-effect hypothesis posits that the loss of interest would attenuate the motivational impact of the remaining incentives, as its absence is directly contrasted with prior presence, triggering a negative RPE that devalues the subjective utility of the incentive. In contrast, the transfer-effect hypothesis proposes that prior high interest replenishes cognitive and emotional resources that sustain persistence even after interest diminishes, thereby maintaining or at least delaying the decline in the effectiveness of subsequent incentives.

**Fig. 1:**
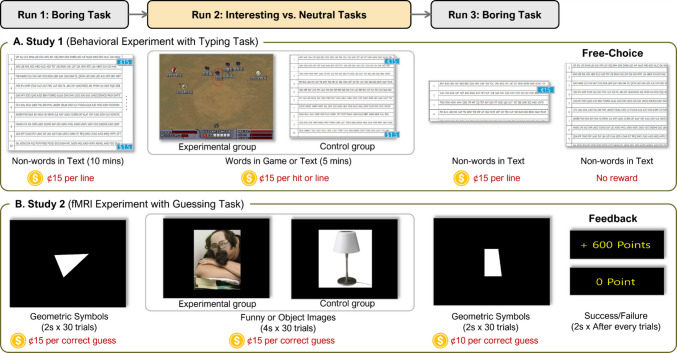
Experimental procedure and materials used in Studies 1 and 2. In both studies, the experimental group completed a Boring–Interesting–Boring task sequence, while the control group followed a Boring–Neutral–Boring sequence. **A.** In Study 1, participants performed a typing task under two of the following three conditions: boring (nonsense letter clusters in text format), neutral (real words in text format), and interesting (real words in a game-like format). Monetary rewards were provided in all conditions. Following the final run, participants entered a free-choice period where they could continue the task without additional incentives. **B.** In Study 2, participants engaged in a social preference guessing task during fMRI scanning, under two of three conditions: boring (geometric symbols), neutral (everyday objects), and interesting (humorous images). Bogus feedback was used to ensure balanced success and failure rates. In Run 3, the magnitude of monetary rewards was reduced to examine sustained engagement under diminished extrinsic incentives. (Color figure online)

To test these ideas, we conducted two complementary studies. Figure 1 presents the overall experimental paradigms of the two studies. Study 1 employed a behavioral typing task to assess changes in effort, motivation, and persistence. Study 2 used fMRI with a social preference guessing task to examine neural mechanisms. In both studies, participants completed two sessions of a boring, incentivized task. Between sessions, the experimental group performed an interesting version of the task (boring–interesting–boring), whereas the control group completed a neutral version (boring–neutral–boring) with matched incentives. In Study 2, monetary incentives were reduced in Run 3 to test whether motivational benefits from prior interest exposure would persist under lower monetary reward conditions.

If the contrast-effect hypothesis were true, we expected the experimental group in Study 1 to show lower effort, interest, willingness to reengage, performance, and free-choice engagement after the interesting element was removed (Run 3) than the control group, reflecting a devaluation of the task following the removal of interest. In Study 2, we predicted increased activation in regions associated with the mesocorticolimbic dopaminergic pathway (VTA, striatum, ACC, vmPFC, amygdala) and the salience network (particularly AI) during Run 2 (with interest), followed by a decline in Run 3 (after removal). Such results would mirror the undermining effect. Within the dopaminergic pathway, we particularly expected activation in the VTA and striatum, as these regions play central roles in regulating reward learning and effort (Salamone & Correa, 2024) and were identified in a prior neuroimaging study on the undermining effect (Murayama et al., 2010).

If the transfer effect hypothesis were true, we predicted the opposite pattern. In Study 1, the experimental group would demonstrate greater effort, interest, willingness to reengage, performance, and free-choice engagement in Run 3 relative to the control group. In Study 2, we anticipated sustained or even elevated activation across Runs 2 and 3 in mesocorticolimbic and salience-network regions compared with Run 1, reflecting a lingering motivational benefit from prior intrinsic engagement. This prediction rests on the idea that during the interesting phase, motivation temporarily shifts from incentives toward the task itself, with dopaminergic responses tracking interest rather than incentive value. Once the interesting element was removed, dopaminergic responses would return to encoding the incentive; however, this temporary shift could replenish motivational systems, enhancing the salience and subjective value of subsequent rewards.

## Study 1

Study 1 employed a behavioral experimental paradigm to examine the role of interest in extrinsically rewarded boring tasks. Participants completed a monetary-incentivized typing task across three runs: a *boring–interesting–boring* sequence for the experimental group and a *boring–neutral–boring* sequence for the control group. We assessed participants’ self-reported effort expenditure, interest, willingness to reengage with the task, and objective task performance. At the end of the experiment, we also recorded participants’ free-choice engagement as an additional indicator of voluntary effort.

### Method

#### Participants

Forty-six undergraduate students (31 women, 15 women) enrolled in an elective educational psychology course participated in the experiment in exchange for course credit. Participants were randomly assigned to either the experimental group (*n* = 23) or the control group (*n* = 23). However, four participants were excluded from the analysis due to technical issues that resulted in missing performance data. The final sample was 22 participants (13 men, seven women) in the experimental group and 20 participants (14 men, eight women) in the control group. Written informed consent was obtained from all participants prior to the study.

#### Materials

Figure 1A illustrates the experimental procedure and materials used for Study 1. In Study 1, we used a typing task that required participants to copy a series of alphabet clusters displayed on a computer screen using a keyboard. Each cluster consisted of three to six letters and was either nonsense strings (e.g., *GKN*, *MGPL*, *HGPO*) or actual words (e.g., *STATE*, *ECHO*, *SIMPLE*). The task was presented in either a standard text-based format or a game-like format. There were three task conditions, which were defined by the type of alphabet clusters and their presentation format: (1) the *boring task* involved copying nonsense clusters in text format, (2) the *neutral task* involved copying actual words in text format, and (3) the *interesting task* involved copying actual words in a game-like format.

Throughout the task, participants earned monetary rewards based on their performance. They were informed in advance that they would receive approximately 15¢ per line typed, with each line consisting of 16 to 18 words. This payment structure remained consistent across all task conditions. The accumulated earnings were displayed on the right side of the screen in the text-based format (boring and neutral conditions). In the game-like format (interesting condition), the total number of successful hits was instead shown at the bottom of the screen to reinforce the gaming experience.

#### Procedure

All participants individually completed three experimental runs, with task sequences varying by group (see Fig. 1). In Run 1, participants in both groups completed the same boring task, which involved copying nonsensical alphabet clusters in a standard text format for 10 min. In Run 2, task conditions diverged between groups. The control group completed a neutral task, a slightly modified version of the boring task, in which they copied real words in a standard text format. Meanwhile, the experimental group completed the interesting task, where they copied real words presented in a game-like format. Both groups had a 5-min time limit for this run. In Run 3, all participants returned to the original boring task and were asked to type four lines with no time limit. The time taken to complete Run 3 was recorded. At the end of each run, participants rated the perceived interestingness of the task they had just completed. After Run 3, they also reported their perceived effort expenditure during Run 3 and their willingness to reengage in the task.

Following the three task runs, participants were told that the experimenter would step out to finalize and retrieve the monetary incentives they had earned during the task, thereby creating a free-choice period. During this time, they were free to continue the task for as long as they wished—or to do anything else—without any time constraints or additional monetary incentives. The task allowed for a maximum of 51 lines, with participants completing anywhere from 0 to 51 lines. Finally, they were debriefed and thanked for their participation.

#### Measures

We utilized three self-report measures in Study 1: perceived task interestingness, effort expenditure, and willingness to reengage in the task. Participants responded to all survey items using a 7-point Likert scale, ranging from 1 (*not true at all*) to 7 (*very much true*). Perceived task interestingness was assessed after each run with a single item measure asking participants to rate how interesting they found the task (e.g., “Please rate the perceived interestingness of the first task run”). We used a single item to measure perceived task interestingness in order to minimize participant fatigue, given that participants were asked to rate the interestingness of three separate tasks. The item we selected was concise, easy to understand, and demonstrated high face validity as a direct measure of perceived task interestingness, which are important criteria for an effective single-item measure (Allen et al., 2022). Effort expenditure was assessed with four items evaluating participants’ effort during Run 3 (e.g., “Even when the task felt boring, I put in my best effort.”; α =.76; Elliot et al., 1999). Willingness to reengage in the task was measured using three items (e.g., “I would be willing to participate in this task again.”; α =.89; Woo et al., 2014).

In addition, three behavioral measures were computed: performance in Runs 1 and 3 and free-choice engagement. Performance was calculated as the number of lines typed divided by the total time spent (in minutes) on each task run (Performance = lines/time). In Run 1, performance was calculated by dividing the number of lines typed by 10 min. In Run 3, performance was calculated by dividing the four lines typed by the time taken to complete them. Since Run 2 involved different task formats across groups, its performance scores were not directly comparable to Runs 1 and 3 or between groups; therefore, Run 2 performance data were excluded from further analysis. Finally, free-choice engagement was quantified by counting the total number of lines participants voluntarily typed.

### Results and discussion

#### Manipulation check

Descriptive statistics for all variables included in the analysis are presented in the upper section of Table 1. To verify the effectiveness of the task interestingness manipulation, participants’ ratings of perceived task interestingness for Runs 1 and 2 were compared. An independent samples *t* test revealed no significant difference between groups in perceived task interestingness during Run 1, when both groups completed the same boring task (*M*s = 3.23 for the experimental group and 3.00 for the control group), *t*(40) =.52, *p* >.05. This indicates no baseline differences in interest between the groups. In contrast, during Run 2, participants in the experimental group, who engaged in the interesting task, rated the task as significantly more interesting than participants in the control group, who completed the neutral task (*M*s = 5.95 and 4.55, respectively), *t*(40) = 5.09, *p* <.001. These results confirm that the experimental manipulation successfully increased perceived task interestingness in the experimental group compared with the control group.

**Table 1:** Descriptive statistics and group differences in measured variables from Studies 1 and 2

Variable	Possible range	α	Experimental group		Control group		*t*
			*M*	*SD*	*M*	*SD*	
**Study 1 (*n*s = 22, 20)**							
Run 1 Interestingness	1–7	–	3.23	1.54	3.00	1.26	.52
Run 2 Interestingness	1–7	–	5.95	.79	4.55	1.00	5.09^***^
Run 3 Interestingness	1–7	–	3.23	1.27	2.60	1.43	1.51
Run 3 Effort Expenditure	1–7	.76	5.06	1.24	4.45	1.20	1.61
Willingness to Reengage	1–7	.89	5.02	1.73	3.68	1.14	2.92^**^
Run 1 Performance	–	–	1.93	.54	1.58	.53	2.11^*^
Run 3 Performance	–	–	2.03	.79	1.50	.50	2.58^*^
Free-choice Engagement	0–51	–	24.81	18.48	19.23	13.67	1.10
**Study 2 (*n*s = 24, 19)**							
Run 1 Interestingness	1–7	–	4.25	1.48	4.32	1.29	−.15
Run 2 Interestingness	1–7	–	5.96	.69	5.21	1.08	2.75^**^
Run 3 Interestingness	1–7	–	4.17	1.31	4.11	1.37	.15
Willingness to Reengage	1–7	.89	4.49	1.52	4.28	1.25	.48

In Study 1, the experimental group *n* = 22, the control group *n* = 20. In Study 2, the experimental group *n* = 24, the control group *n* = 19. In Study 1, performance was measured as the number of lines typed per minute, while free-choice engagement was assessed based on the total number of lines typed. The *t* statistic represents the results of an independent-samples *t* test. ^*^p <.05, ^**^p <.01

#### Group differences in effort, engagement, and performance

Next, we examined group differences in participants’ self-reported interest, effort expenditure, and willingness to reengage in the task during Run 3, as well as their Run 3 performance and free-choice engagement. Although participants were randomly assigned to groups, a significant difference emerged in their performance during Run 1. Specifically, participants in the experimental group typed significantly more lines than those in the control group within a 10-min period during Run 1 (*M*s = 1.93 and 1.58, respectively), *t*(40) = 2.11, *p* =.04. This result suggests a preexisting difference in typing competence between the groups. To account for this discrepancy, Run 1 performance was controlled for in all subsequent analyses. There were no significant gender differences in measured variables.

**Fig. 2:**
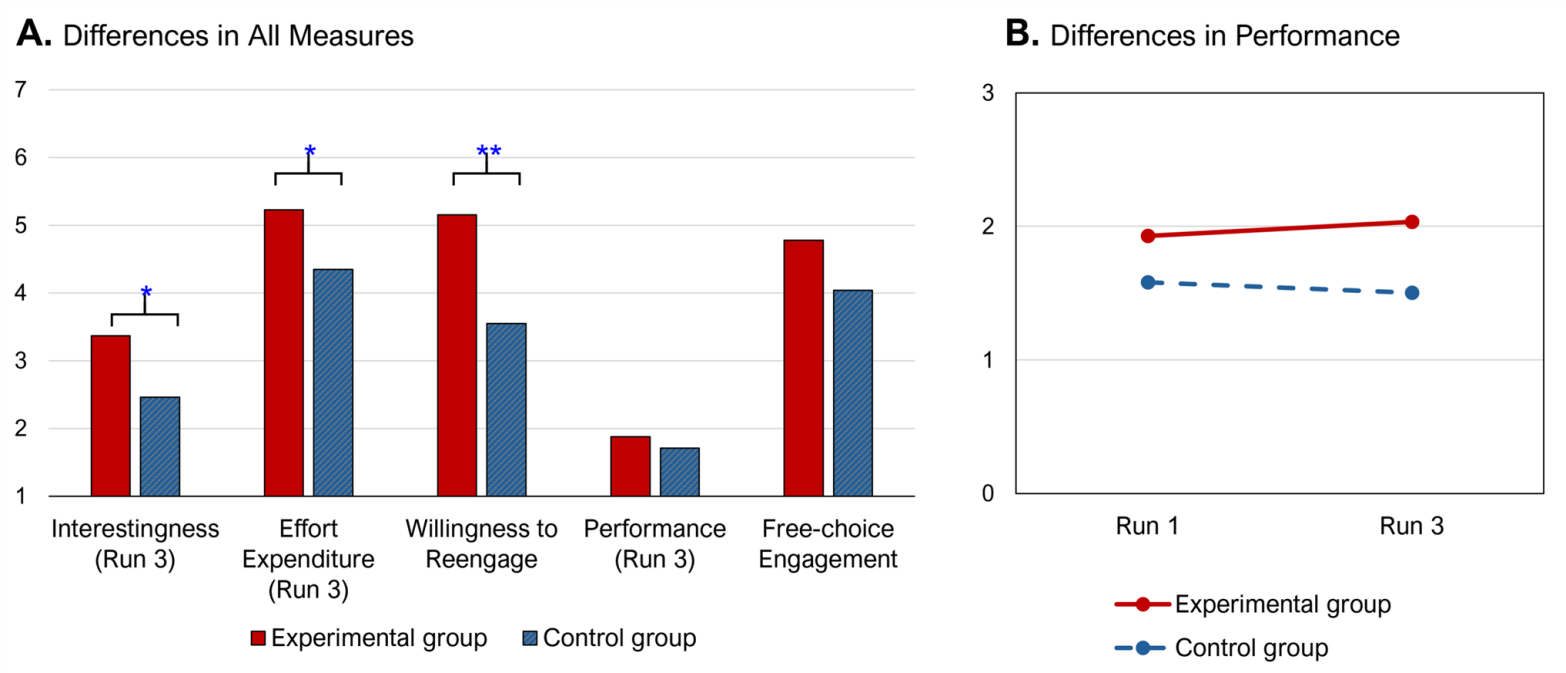
Group differences in measures from Study 1. **A.** Results from a MANCOVA comparing self-reported interest, effort expenditure, willingness to reengage, performance, and free-choice engagement between groups, controlling for Run 1 performance. Free-choice engagement was rescaled by dividing the raw number of voluntarily typed lines by 5 to align visually with other measures. **B.** A 2 × 2 repeated-measures ANOVA examining performance across Runs 1 and 3. The interaction effect was marginally significant (*p* =.089). ^*^p <.05, ^**^p <.01. (Color figure online)

A multivariate analysis of covariance (MANCOVA) was conducted to examine differences in interest, effort expenditure, willingness to reengage in the task, performance, and free-choice engagement, with group as a fixed factor and Run 1 performance as a covariate. The results indicated that the experimental group reported significantly higher levels of Run 3 interestingness, *F*(1, 40) = 4.16, *p* =.048, Run 3 effort expenditure, *F*(1, 40) = 4.85, *p* =.03, and willingness to reengage, *F*(1, 40) = 10.65, *p* =.002, than the control group (Fig. 2A). However, no significant group differences were observed for Run 3 performance or free-choice engagement, despite their mean values being higher in the experimental group compared with the control group.

To further investigate performance trends across task runs, a 2 × 2 repeated-measures ANOVA was conducted with run (Run 1 vs. Run 3) as a within-subject variable and group (experimental vs. control) as a between-subject variable. Results demonstrated a marginally significant interaction effect, *F*(1, 40) = 3.05, *p* =.089. The interaction pattern, as shown in Fig. 2B, indicates that while the control group’s performance exhibited a slight decline from Run 1 to Run 3, the experimental group’s performance showed a modest improvement over the same period.

The findings from Study 1 provided support for the transfer effect hypothesis. Participants who engaged in an interesting task between two boring, but incentivized tasks reported greater interest and exerted more effort during the second boring task (Run 3) compared with those who engaged in a neutral task in between. Although the interaction effect for performance was only marginally significant, participants in the experimental group showed an improvement from Run 1 to Run 3, whereas those in the control group experienced a decline. Additionally, the experience of the sandwiched interesting task significantly increased participants’ willingness to reengage in the task upon completion. A trend toward greater voluntary engagement during the free-choice period was also observed. Overall, these findings align with the idea that intermittent exposure to interesting tasks can act as a motivational boost, replenishing participants’ energy and sustaining their effort and engagement in otherwise monotonous tasks.

## Study 2

Study 2 aimed to replicate the behavioral effects observed in Study 1 while examining the underlying neural mechanisms. The experimental structure closely mirrored that of Study 1, with two key differences. First, participants performed a social preference guessing task, in which they predicted others’ preferences based on visual stimuli to earn monetary rewards. This change was intended to ensure fMRI compatibility. Second, the magnitude of the monetary incentive was reduced in Run 3 compared with Runs 1 and 2, allowing us to examine whether the motivational effects of interest would persist even under diminished incentives.

### Method

#### Participants

A total of 45 right-handed college students (23 men, 24 women; mean age = 22.2 years, *SD* = 2.25) were recruited for this study via online postings. All participants underwent a brief screening interview to ensure they had no history of psychiatric or medical conditions. The study protocol was approved by the university’s institutional review board, and all participants provided written informed consent prior to the scanning session. Each participant received approximately $25 as compensation for their participation.

Participants were randomly assigned to either the experimental group (24 participants) or the control group (21 participants). Two participants were excluded from all subsequent analyses due to excessive head motion (> 3 mm in any direction). As a result, data from 43 participants (24 in the experimental group, mean age = 22.12, 11 men, 13 women; and 19 in the control group, mean age = 22.0, 9 men, 10 women) were included in the final analysis.

#### Materials

Figure 1B illustrates the experimental procedure and materials used for Study 2. Study 2 employed a social preference guessing task, where participants predicted how their peer college students evaluated presented stimuli. They were informed that the stimuli had been assessed by over 500 fellow college students. Their task was to determine whether each stimulus had been judged favorably or unfavorably by the majority of these students.

As in Study 1, the task consisted of three conditions—boring, neutral, and interesting—and participants earned monetary rewards based on their performance. The three conditions differed in the type of stimuli participants were required to judge. For the boring task condition, participants viewed 30 images of simple geometric symbols, such as triangles and circles, displayed in white against a black background. This design was intended to create a dull and unengaging visual experience. For the neutral task condition, participants were shown 30 images of everyday objects, such as a box, vase, ramp, and table.

In the interesting task condition, participants viewed 30 images with humorous content, selected through a pilot survey that confirmed their interestingness even in the absence of monetary incentives. Initially, 74 funny images were sourced from the internet, and 45 college students rated them on a 7-point Likert scale based on perceived interestingness. The 30 highest-rated images, including memes and screenshots from comedy shows, were chosen for the study. The mean interestingness score for the selected images was significantly higher than that of the non-selected images (*M*s = 3.30 and 2.37, respectively), *t*(72) =  − 11.47, *p* <.05. It is important to note that both neutral and interesting conditions, assigned to the control and experimental groups respectively, featured color images to ensure comparable levels of visual arousal, with only the level of interestingness differing between the two conditions.

#### Procedure

Upon arrival at the campus brain imaging center, participants provided written informed consent and received detailed instructions about the experiment, including an overview of the task and the monetary rewards. They then completed a practice session consisting of 30 trials to familiarize themselves with the task.

During the fMRI session, participants completed three consecutive runs, each consisting of 30 trials, resulting in a total of 90 trials. The experimental design of Study 2 closely mirrored that of Study 1. Participants in both the experimental and control groups completed the boring task in Run 1. However, in Run 2, only the experimental group engaged in the interesting task, while the control group completed the neutral task. After Run 2, both groups returned to the boring task in Run 3.

Stimuli in boring condition were presented for 2 s. Due to the complexity of the images in neutral and interesting conditions, stimuli in these conditions were displayed for 4 s. Both the experimental and control groups were instructed to assess other college students’ preferences within the allotted time for each trial. Following their response, feedback about monetary rewards was displayed for 2 s.

Participants were informed in advance that they would receive a performance-contingent monetary reward at the end of the experiment. In Runs 1 and 2, they earned 600 points (approximately 15¢) for each correct guess of college students’ preference, while in Run 3, the reward was reduced to 400 points (approximately 10¢) per correct response. This reduction aimed to examine the role of interest in sustaining engagement with the boring task when incentives were diminished. To ensure a comparable experience of success across participants, bogus feedback was implemented, with the sequence of success feedback counterbalanced. Success and failure were evenly distributed, each occurring 50% of the time.

Following the scanning session, participants completed a self-report questionnaire assessing the perceived interestingness of each run and willingness to reengage in the task. Finally, participants were debriefed and thanked for their participation.

#### fMRI data acquisition and preprocessing

Imaging data were acquired using a 3 T Siemens Trio MRI scanner at the university’s on-campus brain imaging center. For functional imaging, blood-oxygen-level-dependent (BOLD) contrast images were acquired using a single-shot gradient echo planar imaging (EPI) sequence with the following parameters: TR = 2,000 ms, TE = 30 ms, FOV = 240 mm, interleaved acquisition, 33 slices, slice thickness = 4 mm, no gap. The obtained images were preprocessed using SPM 12 software. Specially, functional images were realigned to the first volume, corrected for the slice acquisition time, normalized to EPI templates implemented in SPM 12, and finally spatially smoothed using an 8 mm full width at half maximum (FWHM) isotropic Gaussian kernel.

#### fMRI data analysis

For whole-brain analysis, first-level general linear models (GLMs) were constructed for each participant with nine regressors of interest: (1) three success feedback phases, where participants were informed that they had guessed correctly and earned a monetary reward (one for each run); (2) three failure feedback phases, where participants were informed that they had guessed incorrectly and did not earn a monetary reward; and (3) three task phases corresponding to each run. Response time, inter-stimulus intervals (ISIs), and six motion parameters were included as nuisance regressors.

Following the analytical approach of Study 1, we first conducted whole-brain analyses to examine directional group differences for each run independently during both the feedback and task phases. We then conducted a whole-brain 2 (group: experimental vs. control) × 2 (run: first vs. third) ANOVA to assess interaction effects between group and run across both phases. Unlike Study 1, where Run 2 was excluded due to incomparable characteristics, Run 2 in Study 2 was comparable with Runs 1 and 3. Thus, as an exploratory analysis, we conducted a whole-brain 2 (group) × 3 (run: first, second, third) ANOVA, followed by separate 2 × 2 ANOVAs (Run 1 vs. Run 2; Run 2 vs. Run 3) to further examine interaction effects across phases. Significance was assessed using cluster-level family-wise error (FWE) correction at *p* <.05 with cluster-defining threshold of *p* <.005 (uncorrected). Parameter estimates (beta values) for significant clusters were extracted using the MarsBaR toolbox, averaged across participants within each group and run.

### Results and discussion

#### Manipulation check and differences in self-reported measures

The lower section of Table 1 presents the descriptive statistics for self-reported measures in Study 2. Participants’ ratings of task interestingness for Runs 1 and 2 were compared with examine the effectiveness of the task manipulation. An independent samples *t* test revealed no significant difference in perceived task interestingness for Run 1, when both groups completed the same boring task (*M* = 4.25 for the experimental group, *M* = 4.32 for the control group), *t*(41) =  − 0.15, *p* >.05. In contrast, for Run 2, participants in the experimental group reported significantly higher task interestingness compared with those in the control group (*M*s = 5.96 and 5.21, respectively), *t*(41) = 2.75, *p* =.009. These results confirm that the task manipulation was successful.

Next, we compared participants’ reported interestingness for Run 3 and their willingness to reengage in the task. Although the experimental group reported higher interestingness and reengagement intention than the control group did, the group differences in these variables were not significant. This contrasts with the findings of Study 1. One possible explanation for this disparity is the nature of the tasks. In Study 2, success in guessing other students’ preferences was a matter of chance, which may have introduced uncertainty about the outcome and made the task less tedious. Indeed, participants’ interestingness ratings for Runs 1 and 3 were higher in Study 2 than in Study 1. Despite this, paired *t* tests confirmed that participants in both the experimental and control groups in Study 2 still found Runs 1 and 3 significantly less interesting than Run 2, indicating that these runs remained relatively boring within the study. There were no significant gender differences in measured variables.

#### Results of fMRI data analyses

An exploratory whole-brain 2 × 3 ANOVA examining interaction effects between group (experimental vs. control) and run (first, second, third) across both feedback and task phases revealed no significant clusters survived for the interaction term. This is likely due to the limited statistical power resulting from the small sample size and the relatively few time points in each run. Consequently, we focused on the results of run-by-run group differences using *t* contrasts in both the feedback and task phases, as well as whole-brain 2 × 2 ANOVA (Run 1 vs. Run 3) and exploratory pairwise 2 × 2 ANOVAs (Run 1 vs. Run 2; Run 2 vs. Run 3).

#### fMRI results during the feedback phase

**Fig. 3:**
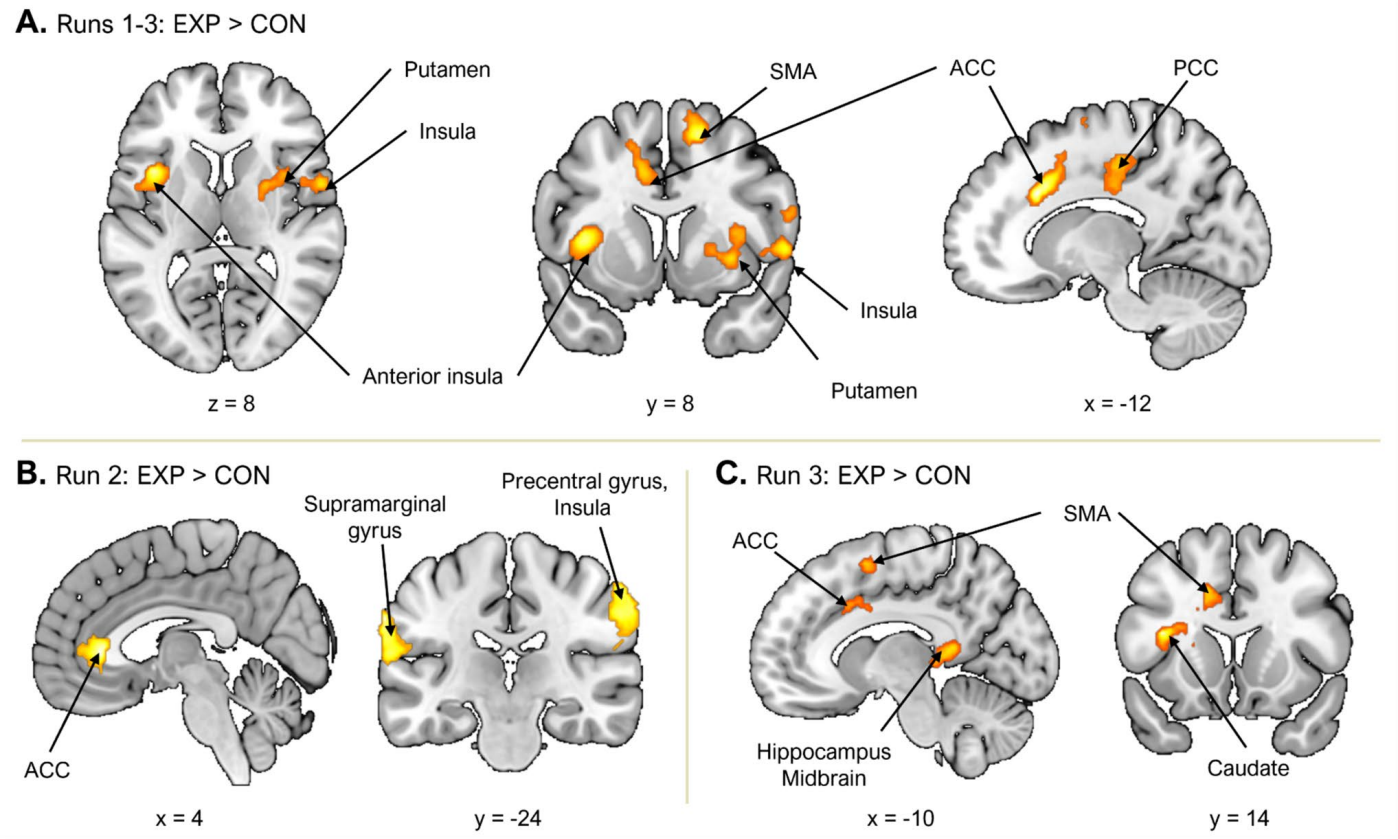
Group differences in brain activations during feedback (Success – Failure) phase of Study 2. Run-by-run comparisons revealed regions showing significantly greater activation in the experimental group compared with the control group. **A.** Regions showing greater activation in the experimental group across Runs 1 to 3. **B.** Regions with significantly greater activation in the experimental group during Run 2. **C.** Regions with significantly greater activation in the experimental group during Run 3. EXP = experimental group; CON = control group; ACC = anterior cingulate cortex; SMA = supplementary motor area; PCC = posterior cingulate cortex. (Color figure online)

The upper section of Table 2 presents significant brain activations observed during the feedback phase (success vs. failure) across runs. Across all three runs, no brain regions exhibited greater activation in the control group compared with the experimental group. In contrast, the experimental group showed greater activation from Runs 1 to 3 in a broad network comprising the bilateral ACC, right supplementary motor area (SMA), bilateral AI, bilateral posterior cingulate cortex (PCC), left supramarginal gyrus, right putamen, right pallidum, and right insula (Fig. 3A). Notably, increased activation in the experimental group began to emerge in Run 2, with elevated responses in the left supramarginal gyrus, right ACC, and right insula during success feedback relative to failure feedback (Fig. 3B). By Run 3, this pattern expanded to include the left AI, left striatum (caudate), left SMA, left ACC, left hippocampus, and left midbrain (Fig. 3C).

**Table 2:** Significant brain activations during the feedback (Success – Failure) Phase in Study 2

Region	Side	Cluster-level statistics		Peak-level statistics			
				*t* value	MNI coordinates		
		Size	*p*(FWE)		*x*	*y*	*z*
**Run-by-Run Comparison**							
Runs 1–3: EXP > CON							
ACC	L	337	.010	5.31	− 12	20	30
SMA	R	618	<.001	4.91	12	0	62
AI	L	286	.027	4.80	− 38	10	8
Inferior temporal gyrus, insula	R	1,102	<.001	4.78	54	− 36	18
PCC	R	488	.001	4.33	12	− 24	42
Supramarginal gyrus	L	320	.014	4.14	− 64	− 22	20
Inferior frontal gyrus, insula	R	394	.004	4.06	58	6	2
Putamen, pallidum, AI	R	260	.043	3.73	32	8	− 2
PCC	L	327	.013	3.60	− 10	− 26	46
Run 2: EXP > CON							
Supramarginal gyrus	L	410	.003	4.68	− 66	− 24	34
ACC	R	284	.028	4.45	4	32	4
Precentral gyrus, insula	R	972	<.001	4.38	42	− 4	44
Run 3: EXP > CON							
AI, striatum (caudate)	L	268	.023	5.43	− 36	14	16
SMA, ACC	L	759	<.001	4.21	0	− 6	76
Hippo, thalamus, midbrain	L	265	.024	3.95	− 10	− 42	8
**2 × 2 ANOVA**							
Group × Run (Runs 1 vs. 3)							
Inferior parietal lobule	L	238	.041	4.11	− 32	− 52	26
Lateral occipital cortex	R	403	.002	4.11	44	− 70	− 8
Cerebellum	R	256	.028	3.65	24	− 76	− 36
Group × Run (Runs 2 vs. 3)							
vmPFC	R	340	.006	4.73	12	58	− 2

Significance was assessed using cluster-level family-wise error (FWE) correction at *p* <.05 with cluster-defining threshold of *p* <.005 (uncorrected). *EXP* experimental group; *CON* control group; *ACC* anterior cingulate cortex; *SMA* supplementary motor area; *AI* anterior insula; *PCC* posterior cingulate cortex; *Hipp *hippocampus; *vmPFC* ventromedial prefrontal cortex

**Fig. 4:**
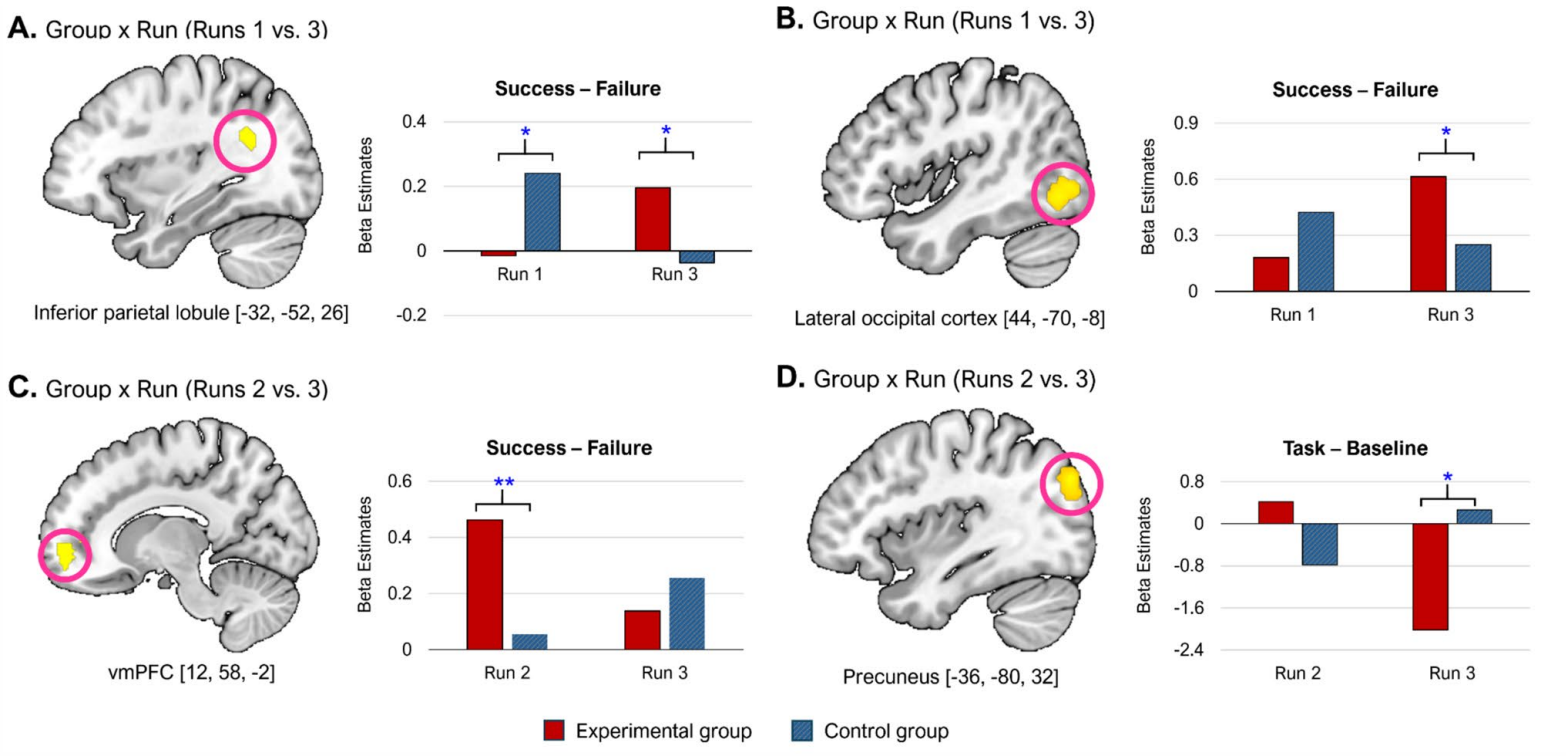
Brain regions showing significant 2 × 2 interaction effects in Study 2. **A.–B.** Left inferior parietal lobule and right lateral occipital cortex showing significant Group × Run (Runs 1 vs. 3) interactions during the feedback phase. **C.** Right ventromedial prefrontal cortex (vmPFC) showing a significant Group × Run (Runs 2 vs. 3) interaction during the feedback phase. **D.** Left precuneus showing a significant Group × Run (Runs 2 vs. 3) interaction during the task phase. ^*^p <.05. (Color figure online)

The lower section of Table 2 reports results from the 2 × 2 ANOVA analyses. A significant interaction between group and run (Run 1 vs. Run 3) were found in the left inferior parietal lobule and right lateral occipital cortex, regions known to support attentional control (Murray & Wojciulik, 2004; Shapiro et al., 2002). While the control group initially showed greater activation in these regions during Run 1, the experimental group exhibited significantly increased activation by Run 3 (Figs. 4A and B). An additional interaction effect between group and run (Run 2 vs. Run 3) was observed in the right vmPFC during the exploratory analysis. As shown in Fig. 4C, the experimental group exhibited significantly higher vmPFC activation than the control group during success feedback in Run 2, although this group difference disappeared by Run 3.

Taken together, the neuroimaging results during the feedback phase provide support for the transfer effect hypothesis. Compared with the control group, the experimental group exhibited enhanced activation across a broader set of regions spanning the mesocorticolimbic dopamine system and the salience network, including the ACC, vmPFC, AI, hippocampus, striatum, and midbrain. These areas have been implicated in effort allocation, effort-based decision-making, motivational salience, and reinforcement learning (e.g., Lopez-Gamundi et al., 2021; Menon & Uddin, 2010; Salamone & Correa, 2024; Treadway et al., 2012). Of particular interest, the vmPFC, a region known to compute the net subjective value of effort (Lopez-Gamundi et al., 2021), showed heightened activation during the success feedback phase in Run 2. Although the between-group difference in the vmPFC activity did not persist into Run 3, the postinterest benefit likely manifested through other reward-related regions (e.g., ACC, AI, striatum). This suggests that the addition of interest may have enhanced the perceived net worth of effortful engagement, even in an extrinsically rewarded context.

Sustained activity in the ACC and AI, along with the co-activation of the striatum and midbrain during Run 3, provides further supports for the transfer effect hypothesis. The ACC plays a central role in reward prediction and the regulation of effortful control (Klein-Flügge et al., 2016; Vassena et al., 2017), while the AI serves as a core hub of the salience network (Menon & Uddin, 2010). Their engagement in the experimental group during Run 3 suggests that motivational salience and effort justification were sustained, even after the removal of interest and the reduction of incentives. In parallel, the co-activation of the striatum and midbrain under diminished incentive conditions indicates that participants continued to integrate prior intrinsically motivated experiences into ongoing effort-reward valuation (Treadway et al., 2012).

#### fMRI results during the task phase

**Fig. 5:**
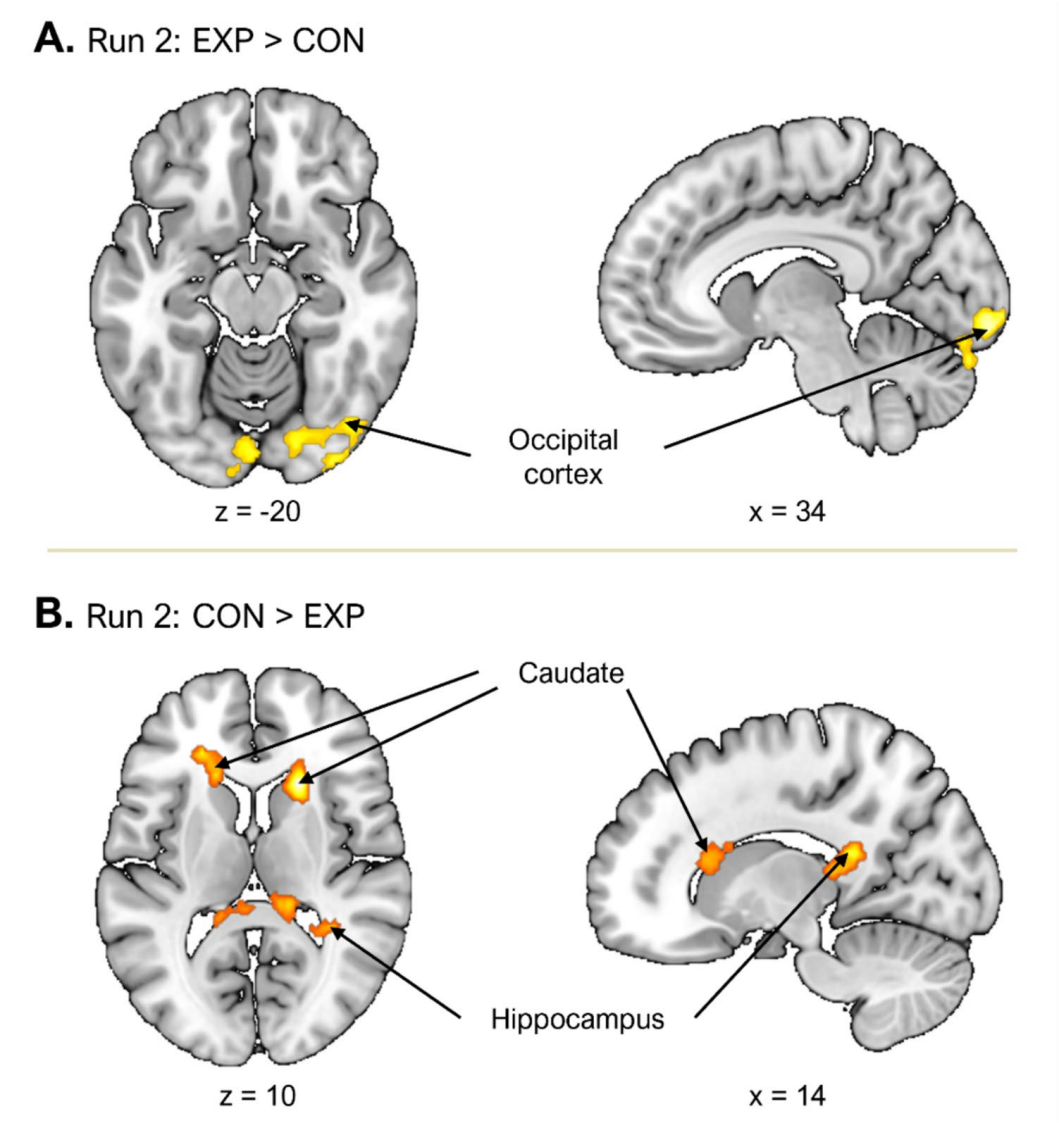
Group differences in brain activations during task (Task – Baseline) phase of Study 2. **A.** Regions with significantly greater activation in the experimental group during Run 2. **B**. Regions with significantly greater activation in the control group during Run 2. (Color figure online)

The upper section of Table 3 presents significant brain activations observed during the task phase (task vs. baseline) across runs. Group differences during this phase emerged exclusively in Run 2. The experimental group showed significantly greater activation in the bilateral occipital cortex compared with the control group (Fig. 5A). The occipital cortex is closely associated with motivated attention, particularly in response to visually salient or emotionally meaningful stimuli. Prior research suggests that activation in this region predicts attention directed toward core life themes (e.g., threat, sex, death), which serve as natural sources of interest (Bradley et al., 2003). This pattern suggests that participants in the experimental group were intensely and intrinsically engaged with the visual content during Run 2. In contrast, the control group exhibited greater activity in the bilateral striatum (caudate), right hippocampus, and right thalamus (Fig. 5B)—regions known to support reinforcement-based, goal-directed, and context-dependent learning (Delgado et al., 2004; Grahn et al., 2008; Iaria et al., 2003) than the experimental group. This was somewhat unexpected, as these regions are typically involved in tracking reward contingencies and reinforcement learning.

**Table 3:** Significant brain activations during the task (Task – Baseline) phase in Study 2

Region	Side	Cluster-level statistics		Peak-level statistics			
				*t* value	MNI coordinates		
		Size	*p*(FWE)		*x*	*y*	*z*
Run-by-Run Comparison							
Run 2: EXP > CON							
Occipital cortex	L	618	<.001	4.01	− 18	− 86	− 20
Occipital cortex	R	324	.012	3.73	34	− 90	− 14
Run 2: CON > EXP							
Striatum (Caudate)	R	374	.005	5.28	20	20	10
Hippo, thalamus	R	499	.001	4.36	14	− 42	18
Striatum (Caudate)	L	516	<.001	4.28	− 18	26	18
2 × 2 ANOVA							
Group × Run (Runs 2 vs. 3)							
Precuneus	L	306	.007	4.90	− 36	− 80	32

Significance was assessed using cluster-level family-wise error (FWE) correction at *p* <.05 with cluster-defining threshold of *p* <.005 (uncorrected). *EXP* experimental group; *CON* control group; *Hipp* hippocampus

One possible explanation for these results is the differences in cognitive strategies employed by the two groups. In the experimental group, the relative absence of hippocampal and striatal activity, combined with heightened occipital activation, suggests a stronger focus on visual processing. Rather than concentrating on optimizing reward outcomes, participants may have been more immersed in the engaging visual content itself—consistent with a state of intrinsic motivation. In contrast, the control group may have engaged more analytically, drawing on object familiarity, evaluating peer preferences, and integrating reinforcement feedback to guide their decisions. Notably, this intrinsically motivated state in the experimental group during Run 2 may have replenished their cognitive resources, enabling them to maintain attentional control and exert sustained effort in Run 3.

Findings from the exploratory interaction analysis support this interpretation. The lower section of Table 3 presents a significant Group × Run (Run 2 vs. Run 3) interaction observed in the left precuneus. As shown in Fig. 4D, the experimental group exhibited a greater reduction in precuneus activity from Run 2 to Run 3 compared with the control group, despite no initial group differences in this region during Run 2. The precuneus is a key region involved in self-referential thought, autobiographical memory, and is a central hub of the default mode network (DMN), which is typically suppressed during focused, externally directed cognitive tasks (Gusnard & Raichle, 2001). The observed reduction in precuneus activity suggests that the experimental group may have entered a more task-focused cognitive state during Run 3, likely reflecting the suppression of DMN activity to allocate greater attentional resources to the task at hand—consistent with the transfer effect hypothesis.

## General discussion

When a task is monotonous but offers guaranteed monetary compensation, is financial reward alone sufficient to sustain engagement and motivation? The present research investigated whether brief exposure to an interesting activity could replenish cognitive and motivational resources in such tasks. Across both behavioral and neuroimaging experiments, we found that strategically timed episodes of interest produced a transfer effect, sustaining motivation and effort beyond the episode itself, even when monetary incentives were reduced. Participants who experienced a short, interest-eliciting interlude demonstrated greater subsequent effort and heightened activation in brain regions associated with attention and reward sensitivity compared with those in a neutral condition, indicating that interest leaves a residual trace on motivational systems.

Incorporating an interest-enhancing activity was found to yield positive effects. In Study 1, after returning to the boring, incentivized task in Run 3, the experimental group reported greater engagement and exerted more effort than the control group, with modest but measurable gains in performance. Trends toward higher reengagement intention and free-choice participation suggest that these participants assigned greater subjective value to the task. These findings align with accounts that interest replenishes self-regulatory resources, counteracting depletion from sustained effort (Endres et al., 2025; Milyavskaya et al., 2021; O’Keefe & Linnenbrink-Garcia, 2014; Thoman et al., 2011). Previous research has primarily examined the effects of interest in the absence of extrinsic rewards, demonstrating that the restorative benefits of interest may operate without external reinforcement (Endres et al., 2025; Milyavskaya et al., 2021; Thoman et al., 2011). Extending prior work, our data indicate that the same restorative effect also elevates perceived task value within a dull, incentive-driven context.

While prior studies have established the motivational benefits of interest, they have provided limited insight into how this effect unfolds at the cognitive and neural levels. Building on this gap, our findings point to a potential underlying mechanism for the observed transfer effect. The restorative function of interest may reflect a temporary reorientation of attention from external incentives to the task itself. This attentional shift may allow individuals to regain focus and subsequently renew the perceived salience of incentives when they return to the otherwise monotonous, only monetarily rewarded task. In Study 2, the interesting task (Run 2) elicited heightened activation in the occipital cortex in the experimental group during the task phase compared with the control group, which may suggest a transient shift of attentional resources toward the task content (Bradley et al., 2003) and away from exclusive focus on incentives.

The experimental group also exhibited distinct neural responses during the success feedback phase. Across runs, they showed sustained activation of the ACC, potentially reflecting ongoing effort–reward integration as the task was reappraised as more worthwhile. This enhanced reward valuation was further evidenced by greater vmPFC activation in Run 2 and heightened AI activation in Run 3—both regions implicated in reward valuation and salience processing. In addition, during the feedback phase of Run 3, the experimental group demonstrated stronger activation in regions associated with attentional control and reinforcement learning, including the lateral occipital cortex, inferior parietal lobule, hippocampus, and midbrain. Together, these patterns may imply that elevated reward valuation was accompanied by greater attentional allocation to feedback and more effective updating of reward predictions. Notably, these effects persisted into Run 3 despite reduced monetary incentives, indicating a carryover effect of task interest in neural activity.

In contrast, during the feedback phase of Run 3, control participants showed reduced activation in the lateral occipital cortex and inferior parietal lobule—regions involved in attentional processing—suggesting a decline in the salience of monetary incentives. This attenuation was accompanied by less suppression of the precuneus, a core hub of the default mode network (Fransson & Marrelec, 2008), relative to the experimental group during the task phase of Run 3, likely indicating greater task-unrelated thought and the experience of boredom (Danckert & Merrifield, 2018; Raffaelli et al., 2018). These patterns may suggest that prolonged exposure to monetary incentives could lead to desensitization, diminishing their motivational potency over time.

These results can be interpreted within the frameworks of reinforcement learning and RPE, while also extending them by showing that interest can modulate the valuation of subsequent rewards. For control participants, repeated exposure to predictable incentives likely produced no RPE (reward matches expectation) or negative RPE, thereby reducing dopaminergic activity and undermining motivation for monotonous task. By contrast, for the experimental group, dopaminergic activity during the interesting task may have been driven more by task engagement than by incentives, effectively resetting the reward baseline. Upon returning to the monotonous, incentive-driven task, this replenished reward system may have rendered incentives more salient, thereby delaying motivational decline. This interpretation also aligns with previous work demonstrating that interest replenishes self-regulatory resources (Endres et al., 2025; Milyavskaya et al., 2021; O’Keefe & Linnenbrink-Garcia, 2014; Thoman et al., 2011). Overall, our findings suggest that the benefits observed here reflect not merely a transient boost in engagement but a short-term form of motivational self-regulation, in which prior interest enhances the perceived value of subsequent monetary rewards and sustains effort.

Our findings further suggest that rewards and interest are not confined to a trade-off relationship, but can operate synergistically under certain conditions. Prior work has proposed that extrinsic rewards can serve as an effective entry point for engagement, particularly when individuals lack initial motivation to participate (Bardach & Murayama, 2025; Hidi & Harackiewicz, 2000). Once interest is sparked, incentives may be gradually reduced, allowing attention to shift toward the inherent value of the task. In this light, Bardach and Murayama (2025) argued that intrinsic and extrinsic motivation should not be treated as mutually exclusive or inherently good or bad; rather, a more strategic and integrative approach is needed to harness both forms of motivation for optimal outcomes. Our study provides empirical support for one such strategy and further suggests that complete removal of extrinsic rewards may not be necessary (e.g., the workplace). Instead, brief episodes of interest can be embedded within externally incentivized tasks to replenish motivational resources and sustain, or even enhance, effort and engagement over time, especially in low-stimulation contexts.

### Implications of the study

The central principle emerging from this research is that interest-enhancing components can function as motivational resets: they temporarily redirect attentional and dopaminergic systems from incentives toward the task’s inherent value, thereby replenishing cognitive resources. Once the interest-enhancing activity ends, individuals return to the primary monotonous task with renewed capacity for sustained effort, often perceiving subsequent incentives as more salient. Although our experiments were conducted over a short time frame using novel yet intentionally monotonous tasks with low personal relevance, this principle may have broader applicability.

This principle has clear relevance for real-world settings where monotony is inherent and often unavoidable, such as manufacturing, administrative work, and certain forms of training. In such environments, the repetitive nature of the work can gradually erode attention, deplete self-regulatory resources, and diminish productivity, even when incentives (e.g., salary, bonuses) remain constant. Our findings indicate that these motivational costs can be mitigated by deliberately incorporating brief episodes of interesting engagement into the workflow, without altering overall incentive structures or disrupting the core objectives of the task.

The same approach holds promise in educational settings. Repetitive skill practice, such as language drills, mathematical problem sets, or music scale exercises, is essential for mastery but particularly vulnerable to boredom. Introducing strategically timed, interest-enhancing diversions, such as gamified challenges, real-world applications, or personally meaningful content, within incentivized tasks (e.g., points) may help sustain attention and engagement during such practice (O’Keefe & Linnenbrink-Garcia, 2014).

While our study introduced externally embedded sources of interest that were primarily affective, such as game-like elements and humorous images, a more sustainable and impactful approach may lie in fostering internally generated intrinsic motivation or integrating value-based interest (e.g., personal relevance). For example, Sansone et al. (1992) found that individuals who actively transformed boring tasks into interesting ones exhibited greater persistence over time. However, such motivational self-regulation requires two critical components: a reason to persist and access to effective strategies. In their study, participants reinterpreted mundane tasks as meaningful only when provided with both a compelling rationale (e.g., health benefits) and actionable strategies. Similarly, O’Keefe and Linnenbrink-Garcia (2014) showed that interest most effectively replenished self-regulatory resources when both affective and value-related components of interest were high. These findings suggest that helping individuals recognize the utility or personal relevance of seemingly monotonous tasks and equipping them with concrete tools to enhance engagement may be key to promoting sustained effort and meaningful, self-regulated motivation.

Crucially, the strength of this strategy may lie in preserving the existing incentive framework while adding variability to the intrinsic motivational landscape. By doing so, it minimizes the risk of undermining incentives while leveraging the resource-replenishing benefits of interest-based engagement. Over time, this integrated approach may promote more consistent performance, greater persistence, and improved learning outcomes in contexts where monotony cannot be eliminated but can be strategically managed.

### Limitations and future directions

Several methodological and conceptual constraints should be considered when interpreting these findings. First, the use of different tasks in Studies 1 and 2, necessitated by the need for an fMRI-compatible paradigm, limited direct comparison between behavioral and neuroimaging results. In particular, the simplicity and reduced monotony of the social preference guessing task in Study 2 constrained our ability to assess sustained effort and maintain equivalence with the typing task used in Study 1. To improve comparability, future research should develop structurally and cognitively parallel tasks that can be implemented both behaviorally and in the scanner (e.g., sustained attention or *n*-back paradigms).

Second, future studies should incorporate direct motivational and cognitive indicators to further validate the mechanisms we proposed for the observed neural activity. We interpreted that introducing interest temporarily shifted attentional focus away from incentives toward the task itself, and that this shift subsequently enhanced the perceived salience of incentives when interest was removed. These interpretations could be more rigorously tested using measures such as trial-level attention allocation (e.g., eye-tracking or self-reports of off-task thinking) and behavioral indices of reward valuation and replenished mental resources (e.g., effort-discounting tasks in Run 3; Unsworth et al., 2025). Such measures would provide more converging evidence for the hypothesized transfer of motivational focus and the resource-replenishing function of interest.

Third, replication with larger samples is essential. Although the behavioral effects were consistent, they were modest in magnitude, possibly reflecting either inherent limits to the duration or intensity of interest-based replenishment, or simply the constraints of sample size. Likewise, although several mesocorticolimbic regions were identified in Study 2, key areas such as the VTA and nucleus accumbens (Salamone et al., 2007) were not observed, and some effects failed to survive more stringent analyses, likely due to limited statistical power from the small sample size and restricted trial numbers.

Fourth, future work should extend these findings to more ecologically valid tasks. Our interest manipulation was brief and task-specific, designed to test the hypothesized mechanisms under controlled laboratory conditions. However, it remains an open question whether similar transfer effects emerge in more naturalistic contexts, such as prolonged study sessions, repetitive work routines, or skill training that involve performance-contingent monetary incentives. Embedding interest-enhancing episodes in such contexts would allow stronger inferences about the generalizability and practical significance of our findings.

Finally, future studies should also investigate the optimal timing, frequency, and sequencing of interest-enhancing elements. The effectiveness of embedding interesting activities is likely shaped by task structure, reward magnitude and predictability, individual differences (e.g., baseline interest level, boredom proneness, dopamine receptor density), and contextual demands. Understanding these factors will be essential for designing effective, scalable interventions in educational and workplace settings.

### Conclusion

The present research showed that incorporating interest into monotonous, reward-based tasks could restore cognitive and motivational capacities to sustain effort, with benefits that endure beyond the removal of the interesting element. Across behavioral and neuroimaging studies, these episodes of interest revitalized attention, enhanced reward sensitivity, and supported sustained effort, outcomes that were not achieved by monetary incentives alone. By testing contrast-effect and transfer-effect hypotheses, our findings clarify how reward and interest can interact over time, revealing that strategically timed interest-enhancing elements can serve as motivational refresh points rather than a distraction from reward-based goals. This work advances motivation science by integrating reinforcement learning perspectives with theories of interest and self-regulation, offering an empirically grounded principle for designing interventions that sustain persistence in repetitive or low-stimulation contexts.

## Supplementary Information

Below is the link to the electronic supplementary material.

Supplementary file1 (DOCX 24 kb)

## Data Availability

This study was not preregistered. The data from two studies are publicly available at the following link: https://osf.io/ng7rb/?view_only=aa84d2a707a243c0bf4661beb853ac7a

## Code availability

The custom analysis codes are publicly available at the following link: https://osf.io/ng7rb/?view_only=aa84d2a707a243c0bf4661beb853ac7a
