# Supplementary material for: Neurocognitive effects of interest on reward valuation and effort investment in boring contexts
Source: Cogn Affect Behav Neurosci. 2026 Feb 14;26(2):669–87. doi: 10.3758/s13415-026-01403-7 (PMC13095969; doi:10.3758/s13415-026-01403-7)
Supplement: Supplementary file 1 — Supplementary file1 (DOCX 24 kb) [file 13415_2026_1403_MOESM1_ESM.docx]

**Table S1.**

*Complete List of Items Used in Studies 1 and 2*

| **Variable** | **Measured in** | **Items** |
| --- | --- | --- |
| Perceived task interestingness | Studies 1 and 2 | 1. Please rate the perceived interestingness of the first task run. 2. Please rate the perceived interestingness of the second task run. 3. Please rate the perceived interestingness of the third task run. |
| Effort expenditure | Study 1 | 1. Even when the task (Run 3) felt boring, I put in my best effort. 2. Even when the letters were meaningless, I made a diligent effort to type them accurately and quickly. 3. Regardless of whether or not I liked the materials, I worked my hardest to complete the task. 4. When the letters were challenging, I put in extra effort to type them accurately and quickly. |
| Willingness to reengage | Studies 1 and 2 | 1. I would be willing to participate in this task again. 2. I would recommend this task to my friends. 3. I would be interested in engaging with this task further |

*Note*. All variables were assessed using a 7-point Likert scale.
